# Low- and High-Attenuation Lung Volume in Quantitative Chest CT in Children without Lung Disease

**DOI:** 10.3390/children8121172

**Published:** 2021-12-10

**Authors:** Dimitrios Moutafidis, Maria Gavra, Sotirios Golfinopoulos, Antonios Kattamis, George Chrousos, Christina Kanaka-Gantenbein, Athanasios G. Kaditis

**Affiliations:** 1 Division of Pediatric Pulmonology, First Department of Pediatrics, National and Kapodistrian University of Athens School of Medicine & Agia Sofia Children’s Hospital, 115 27 Athens, Greece; moutafidisdim@gmail.com (D.M.); ckanaka@med.uoa.gr (C.K.-G.); 2 CT, MRI & PET/CT Department, Agia Sofia Children’s Hospital, 115 27 Athens, Greece; mmgavra@yahoo.com (M.G.); sotirisgolfinopoulos@gmail.com (S.G.); 3 Division of Pediatric Hematology-Oncology, First Department of Pediatrics, National and Kapodistrian University of Athens School of Medicine & Agia Sofia Children’s Hospital, 115 27 Athens, Greece; ankatt@med.uoa.gr; 4 University Research Institute of Maternal and Child Health and Precision Medicine, UNESCO, National and Kapodistrian University of Athens, 115 27 Athens, Greece; chrousge@med.uoa.gr

**Keywords:** air-trapping, bronchiolitis obliterans syndrome, CT densitometry, lung hyperinflation

## Abstract

In contrast to studies of adults with emphysema, application of fixed thresholds to determine low- and high-attenuation areas (air-trapping and parenchymal lung disease) in pediatric quantitative chest CT is problematic. We aimed to assess age effects on: (i) mean lung attenuation (full inspiration); and (ii) low and high attenuation thresholds (LAT and HAT) defined as mean attenuation and 1 SD below and above mean, respectively. Chest CTs from children aged 6–17 years without abnormalities were retrieved, and histograms of attenuation coefficients were analyzed. Eighty examinations were included. Inverse functions described relationships between age and mean lung attenuation, LAT or HAT (*p* < 0.0001). Predicted value for LAT decreased from −846 HU in 6-year-old to −950 HU in 13- to 17-year-old subjects (cut-off value for assessing emphysema in adults). %TLC_CT_ with low attenuation correlated with age (r_s_ = −0.31; *p* = *0*.005) and was <5% for 9–17-year-old subjects. Inverse associations were demonstrated between: (i) %TLC_CT_ with high attenuation and age (r^2^ = 0.49; *p* < 0.0001); (ii) %TLC_CT_ with low attenuation and TLC_CT_ (r^2^ = 0.47; *p* < 0.0001); (iii) %TLC_CT_ with high attenuation and TLC_CT_ (r^2^ = 0.76; *p* < 0.0001). In conclusion, quantitative analysis of chest CTs from children without lung disease can be used to define age-specific LAT and HAT for evaluation of pediatric lung disease severity.

## 1. Introduction

Quantitative chest computed tomography (CT) has been used extensively to assess the severity of obstructive lung disease in adults [1,2,3]. A low attenuation threshold of −950 HU at full inspiration has been applied in most investigations to identify lung areas affected by emphysema, and by using this threshold it has been shown that CT-quantified disease severity predicts lung function decline [4,5]. Quantitative chest CT has also been utilized in an increasing number of pediatric studies to evaluate the severity of obstructive lung disease such as bronchopulmonary dysplasia, cystic fibrosis, post-infectious bronchiolitis obliterans (PIBO), bronchiolitis obliterans in perinatally HIV-infected adolescents and bronchiolitis obliterans syndrome (BOS) following lung or hematopoietic stem cell transplantation (HSCT), all of which are characterized by air-trapping and volume loss due to atelectasis and fibrosis [6,7,8,9,10,11,12,13,14].

Selection of a specific attenuation coefficient cut-off value expressed in Hounsfield units (HU), below which attenuation measurements correspond to areas of lung hyperinflation, is a major obstacle in the evaluation of pediatric patients with obstructive lung disease by quantitative chest CT. In a retrospective study comparing children with and without history of bronchopulmonary dysplasia, automatic CT segmentation with low attenuation thresholds of −950 UH, −910 UH or −856 HU that have been previously validated in studies with adults could not distinguish the two groups in terms of the degree of lung hyperinflation [7]. Hence, the application of fixed thresholds in pediatrics is problematic. To overcome this difficulty, Kim et al. have proposed individualized attenuation thresholds for quantification of air trapping in children with BOS after HSCT and PIBO [9,15]. For each patient, the authors averaged two mean attenuation values, one from an area with air-trapping and another one from an area with normal lung tissue, both selected by an experienced pediatric radiologist. This approach has the limitation that selection of an attenuation cut-off value is subjective and probably influenced by the degree of experience of the interpreting radiologist.

The Global Lung Function Initiative (GLI) Network have published spirometry and static lung volumes reference equations and predicted lower limits of normal, demonstrating that TLC (air volume), FVC (air volume) and FEV_1_ (airflow rate) essentially increase in a linear fashion from the age of 3 years to approximately the age of 20 years after controlling for standing height [16,17]. It is reasonable to speculate that analogous changes occur in lung tissue attenuation on chest CT with progressive age. Indeed, Stein et al. have reported that mean lung attenuation in children 0–7 years old with normal chest CT decreases rapidly in the first 2 years of life and more slowly from 3 to 7 years, a finding consistent with growing air component relative to tissue component in the lungs [18]. Therefore, the aim of the present investigation that included chest CT examinations without abnormalities in children ≥6 years old was to assess the effects of age on: (I) total lung capacity measured by CT (TLC_CT_); (II) mean lung attenuation at full inspiration; and (III) low and high attenuation thresholds defined as mean and one standard deviation below or above the mean lung attenuation, respectively. We have hypothesized that percent proportions of TLC_CT_ (%TLC_CT_) with low or high attenuation are significantly associated with age and TLC_CT_.

In addition, we aimed to compare our results with findings of a recently published retrospective study by Barrera et al which included pediatric patients with non-contrast-enhanced chest CT and evidence of normal lung parenchyma [19]. Chest CTs were analyzed using fixed low and high attenuation thresholds similar to investigations in adults [19]. Although the type of scanner and settings during image acquisition as well as the image reconstruction algorithm were not identical in the present study and in the investigation by Barrera et al., there were interesting similarities in the results of the two reports, such as, for example, comparable mean lung attenuation values at various ages. There were also remarkable differences like in the type of regression model that describes the relationship between TLC_CT_ and age.

## 2. Materials and Methods

### 2.1. Subjects

The protocol of this cross-sectional study was approved by the Agia Sofia Children’s Hospital Scientific Council (14779/23−06−2017; approved on 19 July 2017). Non-enhanced chest CTs from children with ages ≥6 years that: (I) were performed from January 2016 till July 2018, and (II) were interpreted as “without abnormal findings” were retrieved from the Radiology Department Picture Archiving and Communication System (PACS). Children aged 6 years and above were more likely to follow commands and perform inspiratory breath hold compared with younger subjects. Chest CT exams were obtained in children who were not diagnosed clinically with lung disease, but who underwent the examination for other reasons, such as trauma, mediastinal widening on chest X-ray or neoplastic disease. For each age group (i.e., 6-year-old children, 7-year-old children, etc.) the first 8 examinations in chronological order were retrieved if this number of studies was available. This specific number was selected because after an initial review, it was found that there were at least 7–8 examinations for most age groups during the study period.

### 2.2. Image Acquisition, Analysis, and Definition of Attenuation Thresholds

Non-enhanced chest CT scans were obtained in a BrightSpeed 16 RT multidetector scanner with AW VolumeShare 4 workstation (General Electric Healthcare, Chicago, IL, USA). Non-sedated and spontaneously breathing patients were coached by the radiology technician to hold their breath as close to full inspiration (Total Lung Capacity-TLC) as possible during image acquisition. Beam current ranged from 29 to 210 mA, tube potential from 100 to 120 kV and CT dose index (CTDI) from 1.64 to 9.33 mGy. For 12 subjects CTDI values reflect radiation exposure for combined chest and abdominal CT exams. Automated tube current modulation was used, and the tube voltage (kilovolts) settings were selected based on patient’s weight. Noise index was 11.57. Scanner settings included: slice collimation 0.62 mm, slice thickness 5 mm, gantry rotation time 500 ms, pitch 1.375 and temporal resolution 741 ms. A soft General Electric reconstruction kernel was used to avoid overestimation of the lung volume with low attenuation, and reconstruction slice thickness was 1.25 mm.

**Figure 1:**
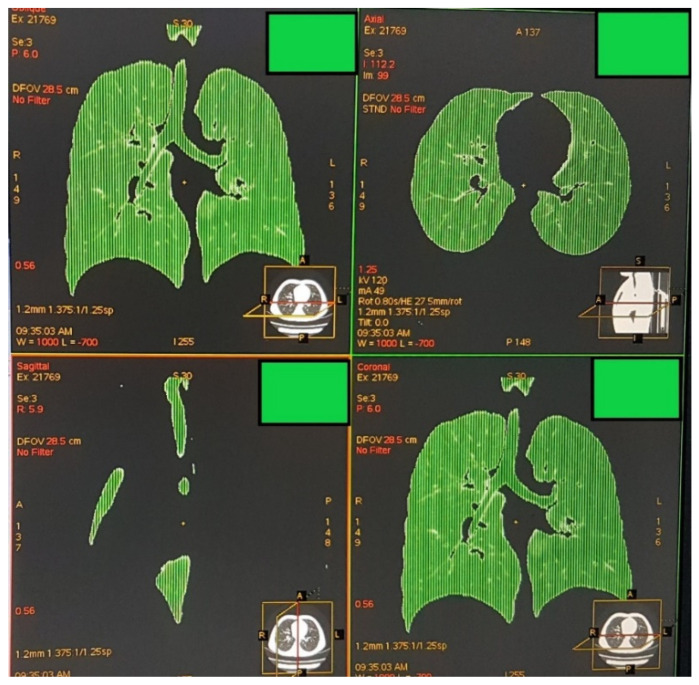
“Lung” was chosen as the imaging window so that other structures were not shown. Air located outside the chest (e.g., gastric air bubble) and outside the subject’s body was still visible and included in the automatically derived attenuation measurements. Thus, it was removed manually, and at the end of the process, only the lung parenchyma, the trachea and bronchi were depicted.

Quantitative image analysis was completed on the acquisition scanner software (AW VolumeShare 4, Version AW4.5_06.022_CTT_5.X; General Electric). On the volume viewer mode, a histogram illustrating frequencies of the attenuation coefficients of all voxels included in acquired images was constructed (see Online Supplementary Methods and Results). On the screen, an axial, a sagittal and a coronal image of the chest were placed. Using two lines vertical to the *x*-axis of the histogram (attenuation coefficient values) an upper and a lower attenuation limit were set visually so that the lung parenchyma was visible in the three chest images (axial, sagittal and coronal). Then, “lung” was chosen as the imaging window so that other anatomical structures were not shown, and air located outside the chest and the subject’s body was removed manually (Figure 1).

**Figure 2:**
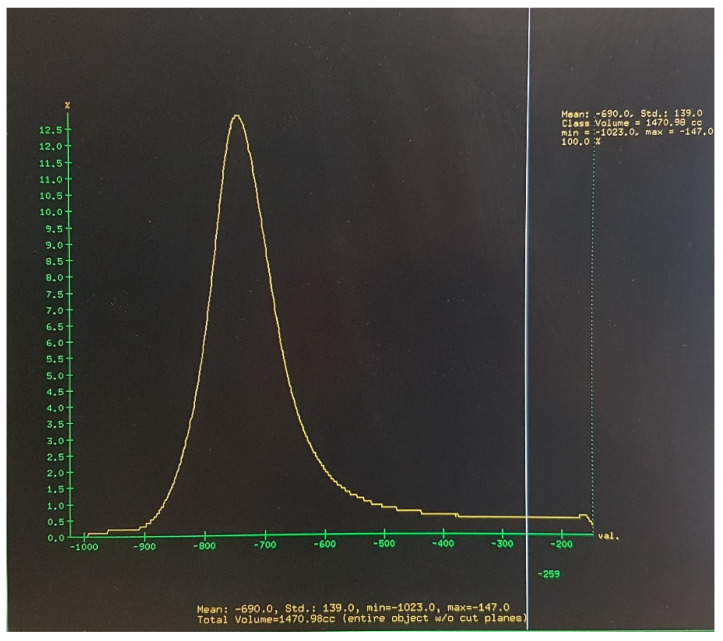
The total lung and airways volume, as well as the minimum attenuation value, maximum attenuation value, and the mean and standard deviation of attenuation values of all voxels included in the images of lung parenchyma and central airways were automatically provided by the software.

The total volume of the lungs and central airways and the mean and standard deviation of the attenuation coefficient values of all voxels contained in images of the lung parenchyma and central airways were automatically calculated by the image analysis software (Figure 2). Air contained in the central airways was trivial (less than 2%) relative to the total calculated lung volume, which was measured practically at full inspiration and corresponded to total lung capacity (TLC_CT_).

A *low attenuation threshold* and a *high attenuation threshold* were calculated for each subject as: (mean attenuation −1SD) and (mean attenuation +1SD), respectively. In addition, %TLC_CT_ with attenuation values < low attenuation threshold (lung volume with attenuation values below (mean attenuation −1SD) divided by TLC_CT_ and multiplied by 100) and %TLC_CT_ with attenuation values > high attenuation threshold (lung volume with attenuation values above (mean attenuation +1SD) divided by TLC_CT_ and multiplied by 100) were calculated. An example of calculation of the low and high attenuation thresholds and %TLC_CT_ with low and high attenuation values is provided in the Online Supplementary Methods and Results.

### 2.3. Outcome Measures and Data Analysis

The primary outcome measures of the study were: (I) %TLC_CT_ with low attenuation, and (II) %TLC_CT_ with high attenuation. Linear regression analysis was completed to assess the association of TLC_CT_ with age, whereas a hyperbola curve (inverse function) was fit to the data to depict the relationship of mean lung attenuation at full inspiration with age. Hyperbola curves were also fit to the data to evaluate the associations between age and low or high attenuation threshold.

Correlations between: (I) %TLC_CT_ with low attenuation or %TLC_CT_ with high attenuation; and (II) age or TLC_CT_ were assessed using the Spearman’s rank correlation coefficient (r_s_). A weak correlation was defined as that with r_s_ > −0.5. To describe the relationship between %TLC_CT_ with high attenuation and age, an inverse function model was fit to the data. Inverse function models were also applied to describe the associations between TLC_CT,_ and %TLC_CT_ with low or high attenuation.

## 3. Results

### 3.1. Subjects’ Characteristics

Between January 2016 and July 2018, 80 non-enhanced chest CTs without abnormal findings from children aged 6 to 17 years (median age 11 years; 40 (50%) female) were retrieved. Image quality of all 80 examinations was adequate for inclusion in the analysis. Subjects’ clinical diagnoses are summarized in Online Supplementary Table S1.

### 3.2. TLC_CT_, Mean Lung Attenuation at Full Inspiration and Their Relationship with Age

For the total group of participants regardless of age, median (minimum, maximum) value of TLC_CT_ was 2794.8 (897.3, 6441.7) mL and median of mean lung attenuation at full inspiration (TLC) was −794.5 (−856, −598) HU.

**Figure 3:**
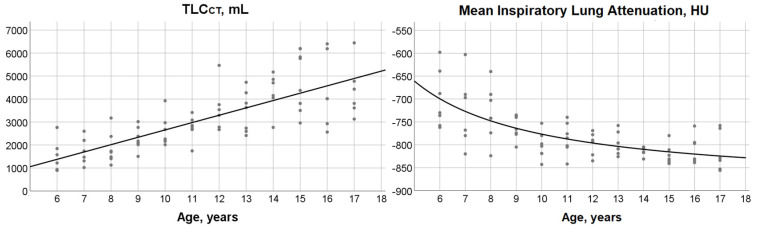
*Left panel:* Scatterplot and fit line depicting the significant linear association between total lung capacity measured by computed tomography scan (TLC_CT_) and age (r^2^ = 0.59; *p* < 0.0001). *Right panel:* Scatterplot and fit hyperbola curve describing the significant relationship between mean inspiratory lung attenuation and age (r^2^ = 0.48; *p* < 0.0001). Dots correspond to measured values for individual subjects.

A significant linear relationship was identified between TLC_CT_ and age:

TLC_CT_ = −541.7 + (319.7 × age), (r^2^ = 0.59; *p* < 0.0001) (Figure 3).

Summary statistics for mean attenuation values at full inspiration according to age group are presented in Table 1. A significant inverse association was demonstrated between mean lung attenuation at full inspiration and age: 

Mean inspiratory lung attenuation = −892.8 + (1160.4/age), (r^2^ = 0.48; *p* < 0.0001; Figure 3). The predicted value of mean inspiratory lung attenuation was −699 HU in the 6-year-old group and decreased to −825 HU at the age of 17 years.

### 3.3. Age-Dependence of Low and High Attenuation Thresholds

**Figure 4:**
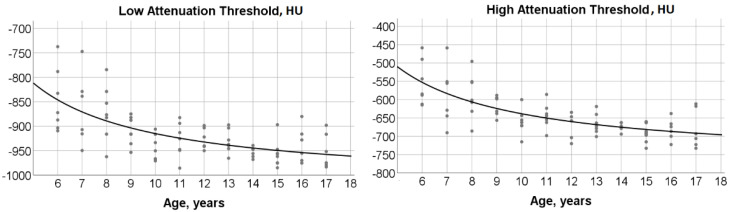
***Left panel****:* Scatterplot and fit hyperbola curve describing the significant relationship between low attenuation threshold and age (r^2^ = 0.42; *p* < 0.0001). ***Right panel****:* Scatterplot and fit hyperbola curve depicting the significant relationship between high attenuation threshold and age (r^2^ = 0.50; *p* < 0.0001). Dots correspond to measured values for individual subjects.

An inverse function described the association between low attenuation threshold and age:

Low attenuation threshold = −1018.5 + (1034.1/age), (r^2^ = 0.42; *p* < 0.0001) (Figure 4).

The predicted value from the equation for low attenuation threshold decreased from −846 HU in 6-year-old children down to approximately −950 HU in 13- to 17-year-old subjects.

Similarly, an inverse function described the relationship between high attenuation threshold and age:

High attenuation threshold = −767.2 + (1286.7/age), (r^2^ = 0.50; *p* < 0.0001) (Figure 4).

Summary statistics for the low and high attenuation thresholds according to age are presented in Table 1.

### 3.4. %TLC_CT_ with Low or High Attenuation and Their Associations with Age and TLC_CT_

Summary statistics for %TLC_CT_ with low or high attenuation in individual subjects according to age and TLC_CT_ range are presented in Table 2.

**Figure 5:**
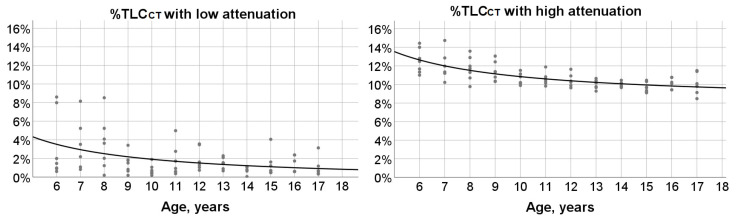
***Left panel****:* Scatterplot outlining the significant, but weak association between % total lung capacity measured by computed tomography scan (TLC_CT_) with low attenuation and age (r^2^ = 0.17; *p* < 0.0001). ***Right panel****:* Scatterplot and fit hyperbola curve describing the significant relationship between %TLC_CT_ with high attenuation and age (r^2^ = 0.49; *p* < 0.0001). Dots correspond to measured values for individual subjects.

A weak significant negative correlation was found between %TLC_CT_ with low attenuation and age (r_s_ = −0.31; *p* = 0.005). %TLC_CT_ with low attenuation was less than 5% in subjects 9–17 years old. A stronger significant negative correlation was identified between %TLC_CT_ with high attenuation and age (r_s_ = −0.67; *p* < 0.0001). An inverse function described the association between %TLC_CT_ with high attenuation and age:

%TLC_CT_ with high attenuation = 8.147 + (26.931/age), (r^2^ = 0.49; *p* < 0.0001) (Figure 5).

**Figure 6:**
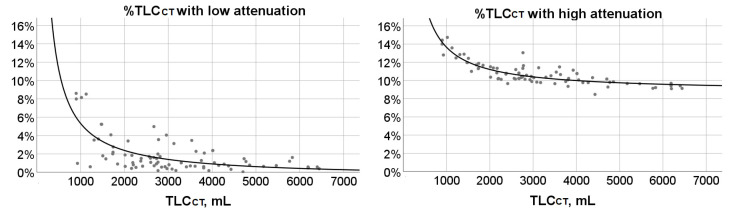
***Left panel****:* Scatterplot and fit hyperbola curve depicting the association between % total lung capacity measured by computed tomography scan (TLC_CT_) with low attenuation and TLC_CT_ (r^2^ = 0.47; *p* < 0.0001). ***Right panel****:* Scatterplot and fit hyperbola curve describing the significant relationship between %TLC_CT_ with high attenuation and TLC_CT_ (r^2^ = 0.76; *p* < 0.0001). Dots correspond to measured values for individual subjects.

Significant correlations were demonstrated between TLC_CT_ and: %TLC_CT_ with low attenuation (r_s_ = −0.50; *p* < 0.0001) or %TLC_CT_ with high attenuation (r_s_ = −0.79; *p* < 0.0001). Inverse functions described the relationship of TLC_CT_ and %TLC_CT_ with low attenuation:

%TLC_CT_ with low attenuation = −0.561 + (5876.6/TLC_CT_), (r^2^ = 0.47; *p* < 0.0001) (Figure 6), or the association between TLC_CT_ and %TLC_CT_ with high attenuation:

%TLC_CT_ with high attenuation = 8.771 + (4875.9/TLC_CT_), (r^2^ = 0.76; *p* < 0.0001) (Figure 6).

## 4. Discussion

In the current report, it has been demonstrated that mean lung attenuation at full inspiration is inversely related to age, whereas similar inverse relationships have been shown for the low and high attenuation thresholds which have been defined for each participant as one standard deviation below or above the mean value, respectively, in the individual histogram of attenuation coefficients. Regression equations for calculating age-specific low and high attenuation thresholds are presented. The predicted value for the low attenuation threshold decreases progressively from −846 HU at the age of 6 years to approximately −950 HU at the ages of 13–17 years, which is the cut-off value commonly selected during processing of CT images in adults with emphysema for the measurement of lung volume affected by air-trapping. Using our proposed definitions of low and high attenuation thresholds, we have shown that %TLC_CT_ with low attenuation remains below 5% in subjects 9–17 years old, whereas %TLC_CT_ with high attenuation diminishes as age and TLC_CT_ increase.

Both in the present study and in the investigation by Stein et al., lung volume estimated by CT increased linearly with age [18]. In this report and due to the inclusion of older subjects who were able to hold their breath at full inspiration, lung volume corresponded to TLC, whereas in the study by Stein et al., which involved mostly infants and preschool children, images were obtained with tidal breathing close to functional residual capacity (FRC) or in other words at 40% TLC. In contrast, Barrera et al. recently merged together data from younger children breathing close to FRC and older children holding their breath at TLC [19]. An exponential function has been used to describe the relationship between calculated lung volume and age instead of two separate linear regression models (one model for FRC vs. age for younger children and one model for TLC vs. age for older children and adolescents) [19]. Of note, the GLI Network have recently published reference equations for static lung volumes [17]. TLC determined by plethysmography or gas dilution techniques increases linearly with age for ages 3 to 20 years, which is consistent with the findings of the present study but not with results of the study by Barrera et al [19].

Mean lung attenuation is inversely related to age. Stein et al. have reported that the attenuation of normal lung parenchyma diminishes from a mean value of −380 HU in neonates down to −650 HU by the age of 2 years [18]. The mean attenuation decline with age is described by a decaying exponential function in this cohort, whereas Barrera et al. have reported a linear relationship between mean lung density and age [18]. In the current report, the predicted value of mean inspiratory lung attenuation was −699 HU in the 6-year-old group and progressively decreased to −825 HU at the age of 17 years. Although the type of relationship between mean lung attenuation in our study and in the report by Barrera et al. differed, mean values for school-aged children and adolescents were remarkably similar in the two investigations (approximately −740 HU and −795 HU, respectively), despite the use of different CT scanners, image acquisition settings and image reconstruction software.

Based on the age-specific low attenuation threshold, a low percentage of voxels with low attenuation coefficients (lower than 5% of TLC_CT_) was identified in subjects 9 years of age or older of this cohort, with a negative relationship between low-attenuation areas and age. In addition, an inverse relationship between %TLC_CT_ with low attenuation and TLC_CT_ was demonstrated. Our results are consistent with findings of published series of adult subjects [20,21]. In a large cohort study including over 5000 adults living in China without emphysema and with normal chest CTs and ages 48–64 years, the upper reference limit (97.5th percentile) for %TLC_CT_ with attenuation coefficients lower than −950 HU was 2.17%, which is comparable to the maximum %TLC_CT_ value of the present report in children aged ≥13 years (2–4%) [20]. In the investigation by Barrera et al., low attenuation areas below −950 HU corresponded to a very low proportion of lung volume [19].

BOS associated with allogeneic HSCT is a serious complication that leads to non-reversible lower airway obstruction, air-trapping and deteriorating lung function [22,23]. We have previously evaluated severity of airway obstruction in children with BOS post-HSCT by quantitative chest CT using an age-specific, low attenuation threshold with a definition identical to the one applied in the present investigation [12]. %TLC_CT_ with low attenuation had a significant decaying exponential association with FEV1/FVC z-score, which is a spirometric index of lower airway obstruction severity.

In a multicenter prospective study in community-dwelling adults, high-attenuation areas on chest CT were defined as those with attenuation coefficients from −600 HU (high attenuation threshold) to −250 HU, and were evaluated as early markers of lung injury predisposing to interstitial lung disease [24]. In the present series of children with healthy lungs, the predicted high attenuation threshold based on the regression equation diminished from −553 HU in 6-year-old children to −692 HU at the age of 17 years. Cryptogenic organizing pneumonia or restrictive lung disease may coexist with BOS in patients who have undergone allogeneic HSCT and reference values for %TLC_CT_ with high attenuation could be useful for evaluating the extent of parenchymal abnormalities [25,26,27]. Indeed, in our previous study, a significant positive association between FEV1/FVC z-score and %TLC_CT_ with high attenuation was identified, which probably reflected coexistence of restrictive lung disease with BOS post-allogeneic HSCT [12].

The strengths of using age-specific attenuation thresholds derived from a cohort of children with healthy lungs are: (I) the threshold selection is objective; (II) measurements can be completed easily using commercial software that accompanies the CT scanner; (III) regression equations can be constructed to calculate predicted values of low and high attenuation thresholds. Changing tube potential settings can alter image quality and affect CT attenuation. More specifically, higher tube potential is related to increased radiation dose and CT attenuation with subsequent improvement in image quality. The use of a low tube potential protocol in chest CT is sufficient for acquiring diagnostic images. We do not expect that a low-dose chest CT protocol will significantly affect the age-dependent low and high attenuation thresholds proposed in this retrospective investigation. For example, in a study of 548 low-dose chest CT examinations in adults without and with COPD, the low attenuation threshold for detection of emphysema did not differ essentially compared with the threshold commonly applied in a conventional higher-dose chest CT (−940 HU vs. −950 HU, respectively).

## 5. Conclusions

Previous authors have proposed subjectively defined, individualized attenuation thresholds for the quantification of air trapping accompanying obstructive lung disease in children, as opposed to fixed attenuation thresholds derived from studies in adult subjects, the use of which is problematic in pediatrics. We have shown that application of age-specific, low attenuation thresholds calculated objectively from chest CTs of children without lung disease provides values of %TLC_CT_ with low attenuation, which are comparable to results of studies including adults with healthy lungs. We suggest that contribution and merging of normal pediatric chest CT quantitative data from pediatric centers around the world could facilitate the calculation of low and high attenuation threshold regression equations for the objective evaluation of lung disease in accordance with the spirometry and static lung volumes reference equations published by the Global Lung Function Initiative Network.

## Figures and Tables

**Table 1:** Summary of mean attenuation values at full inspiration and low and high attenuation thresholds for individual subjects according to age group.

Age Group	Mean Lung Attenuation at Full Inspiration, HU	Low Attenuation Threshold, HU	High Attenuation Threshold, HU
6-year-old (*n* = 7)	−730 (−758, −639) (−762, −598)	−872.3 (−903.5, −788.2) (−909.4, −737.3)	−584.7 (−612.5, −489.8) (−614.6, −458.7)
7-year-old (*n* = 6)	−732.5 (−790, −668.3) (−820, −603)	−872.9 (−924.1, −808.5) (−949.7, −747.1)	−592.2 (−655.9, −528) (−690.3, −458.9)
8-year-old (*n* = 7)	−742 (−774, −690) (−824, −640)	−876.5 (−916.2, −829) (−962.3, −784.3)	−601.5 (−916.2, −829) (−685.7, −495.7)
9-year-old (*n* = 7)	−766 (−777, −738) (−805, −735)	−916.1 (−935.8, −882.1) (−953.5, −874.9)	−597.9 (−637.9, −595.1) (−656.5, −588.3)
10-year-old (*n* = 7)	−803 (−819, −780) (−843, −753)	−950.3 (−967.1, −916.6) (−970.9, −906)	−662.4 (−671.3, −643.4) (−715.1, −600)
11-year-old (*n* = 7)	−785 (−805, −753) (−842, −740)	−925.7 (−949.4, −894.1) (−985.7, −882.4)	−644.3 (−662.3, −623.6) (−698.3, −585.9)
12-year-old (*n* = 6)	−792 (−825.3, −775.8) (−835, −769)	−931.2 (−943.6, −902.1) (−950, −898.6)	−657.7 (−707.8, −643.6) (−720, −634.7)
13-year-old (*n* = 7)	−809 (−819, −772) (−826, −758)	−937.3 (−945.9, −903.6) (−965.4, −897.3)	−672.1 (−686.6, −640.4) (−700.7, −618.7)
14-year-old (*n* = 6)	−812.5 (−820.5, −805) (−831, −805)	−951.7 (−963.5, −945.3) (−968, −939)	−674.1 (−682.5, −662.7) (−694, −662.7)
15-year-old (*n* = 8)	−832.5 (−839, −814) (−841, −780)	−959.4 (−975.4, −949.4) (−985, −897)	−691.8 (−710.7, −668.9) (−732.7, −660.1)
16-year-old (*n* = 6)	−814 (−836, −786) (−839, −759)	−941.8 (−971.5, −907.1) (−975.5, −880.1)	−680.2 (−705.6, −658.9) (−722.6, −637.9)
17-year-old (*n* = 6)	−831.5 (−853.8, −762.5) (−856, −758)	−963.4 (−980.4, −911.9) (−983.3, −898.1)	−699.7 (−725.2, −616.3) (−732.6, −611.5)

Values are expressed as: median (25th percentile, 75th percentile) (minimum, maximum). *Low attenuation threshold* for each subject was defined as: (mean lung attenuation −1SD). *High attenuation threshold* for each subject was defined as: (mean lung attenuation +1SD).

**Table 2:** Values of %TLC_CT_ with low or high attenuation for individual subjects summarized according to age group and TLC_CT_.

Age Group	%TLC_CT_ with Attenuation < Low Attenuation Threshold	%TLC_CT_ with Attenuation > High Attenuation Threshold	TLC_CT_, mL	%TLC_CT_ with Attenuation < Low Attenuation Threshold	%TLC_CT_ with Attenuation > High Attenuation Threshold
6-year-old (*n* = 7)	1.46 (0.90, 7.99) (0.60, 8.60)	12.47 (11.33, 14) (11, 14.43)	897.3–1500 (*n* = 10)	5.23 (2.88, 8.24) (0.60, 8.60)	12.87 (12.35, 14.11) (11.97, 14.73)
7-year-old (*n* = 6)	2.85 (1.02, 5.96) (0.82, 8.15)	11.66 (10.96, 13.32) (10.22, 14.73)	1501–2000 (*n* = 7)	2.01 (1.46, 2.76) (0.90, 4.08)	11.67 (11.30, 11.88) (11.00, 12.43)
8-year-old (*n* = 7)	3.63 (1.22, 5.23) (0.19, 8.52)	11.71 (10.70, 12.89) (9.77, 13.58)	2001–2500 (*n* = 11)	1.05 (0.64, 1.84) (0.42, 3.41)	10.70 (10.18, 11.34) (9.66, 11.51)
9-year-old (*n* = 7)	1.51 (0.64, 1.84) (0.18, 3.41)	11.34 (10.36, 12.43) (10.31, 13.05)	2501–3000 (*n* = 17)	1.50 (0.66, 1.86) (0.18, 4.98)	10.43 (10.18, 11.01) (9.89, 13.05)
10-year-old (*n* = 7)	0.53 (0.40, 1.05) (0.18, 1.89)	10.20 (10.12, 11.13) (9.89, 11.51)	3001–3500 (*n* = 6)	0.71(0.31, 1.56) (0.19, 3.13)	10.34 (9.81, 10.74) (9.77, 11.40)
11-year-old (*n* = 7)	0.92 (0.56, 2.76) (0.35, 4.98)	10.52 (10.13, 10.82) (9.82, 11.88)	3501–4000 (*n* = 9)	0.69 (0.54, 2.19) (0.18, 3.47)	10.23 (9.77, 11.03) (9.35, 11.49)
12-year-old (*n* = 6)	1.45 (0.96, 3.49) (0.73, 3.56)	10.30 (9.82, 11.10) (9.64, 11.63)	4001–4500 (*n* = 7)	0.89 (0.69, 2.37) (0.32, 2.37)	10.07 (9.76, 10.76) (8.47, 10.76)
13-year-old (*n* = 7)	1.5 (0.89, 2.09) (0.66, 2.28)	10.15 (9.66, 10.43) (9.28, 10.65)	4501–5000 (*n* = 4)	0.98 (0.23, 1.40) (0.04, 1.48)	9.83 (9.41, 10.09) (9.28, 10.16)
14-year-old (*n* = 6)	0.77 (0.49, 1.06) (0.04, 1.10)	9.97 (9.74, 10.23) (9.66, 10.45)	5001–5500 (*n* = 2)	0.69 (0.64, 0.73)	9.65 (9.64, 9.66)
15-year-old (*n* = 8)	0.69 (0.48, 1.51) (0.47, 4.10)	9.50 (9.16, 10.16) (9.10, 10.45)	5501–6000 (*n* = 2)	1.41 (1.20, 1.61)	9.18 (9.14, 9.22)
16-year-old (*n* = 6)	1.17 (0.58, 2.37) (0.58, 2.37)	10.23 (9.42, 10.76) (9.42, 10.76)	6001–6500 (*n* = 5)	0.50 (0.43, 0.58) (0.39, 0.58)	9.42 (9.12, 9.56) (9.10, 9.69)
17-year-old (*n* = 6)	0.64 (0.37, 1.66) (0.32, 3.13)	9.94 (8.97, 11.42) (8.47, 11.49)			

Values are expressed as: median (25th percentile, 75th percentile) (minimum, maximum). *Low attenuation threshold* for each subject was defined as: (mean lung attenuation −1SD). *High attenuation threshold* for each subject was defined as: (mean lung attenuation +1SD). TLC_CT_**:** Total lung capacity measured by computed tomography scan. %TLC_CT_ with attenuation values < low attenuation threshold for each subject: Lung volume with attenuation values below (mean attenuation −1SD) divided by TLC_CT_ and multiplied by 100% TLC_CT_ with attenuation values > high attenuation threshold: Lung volume with attenuation values above (mean attenuation +1SD) divided by TLC_CT_ and multiplied by 100.

## Data Availability

Data supporting reported study results are not publicly available.

## Supplementary Materials

Supplementary Methods are available online at https://www.mdpi.com/article/10.3390/children8121172/s1. Figure S1: On the volume viewer mode, a histogram depicting frequencies of the attenuation coefficients of all voxels included in the acquired images of the chest was constructed; Figure S2: An axial, a sagittal and a coronal image of the chest were placed on the screen; Figure S3: “Lung” was chosen as the imaging window so that other structures were not shown; Figure S4: The total lung and airways volume, as well as the minimum attenuation value, maximum attenuation value, and the mean and standard deviation of attenuation values of all voxels included in the images of lung parenchyma and central airways were automatically provided by the software; Figure S5: % total lung volume (%TLC_CT_) with attenuation values < low-attenuation threshold (mean attenuation value −1SD) was calculated; Figure S6: % total lung volume (%TLC_CT_) with attenuation values > high-attenuation threshold (mean attenuation value +1SD) was also calculated; Table S1: Study subjects’ clinical diagnoses.
