# Supplementary material for: Low- and High-Attenuation Lung Volume in Quantitative Chest CT in Children without Lung Disease
Source: Children (Basel). 2021 Dec 10;8(12):1172. doi: 10.3390/children8121172 (PMC8700567; doi:10.3390/children8121172)
Supplement: Supplementary file 1 [file children-08-01172-s001.zip › children-1461287-supplementary.pdf]

children

MDPI

Supplementary Methods

An example of calculation of the low- and high-attenuation thresholds

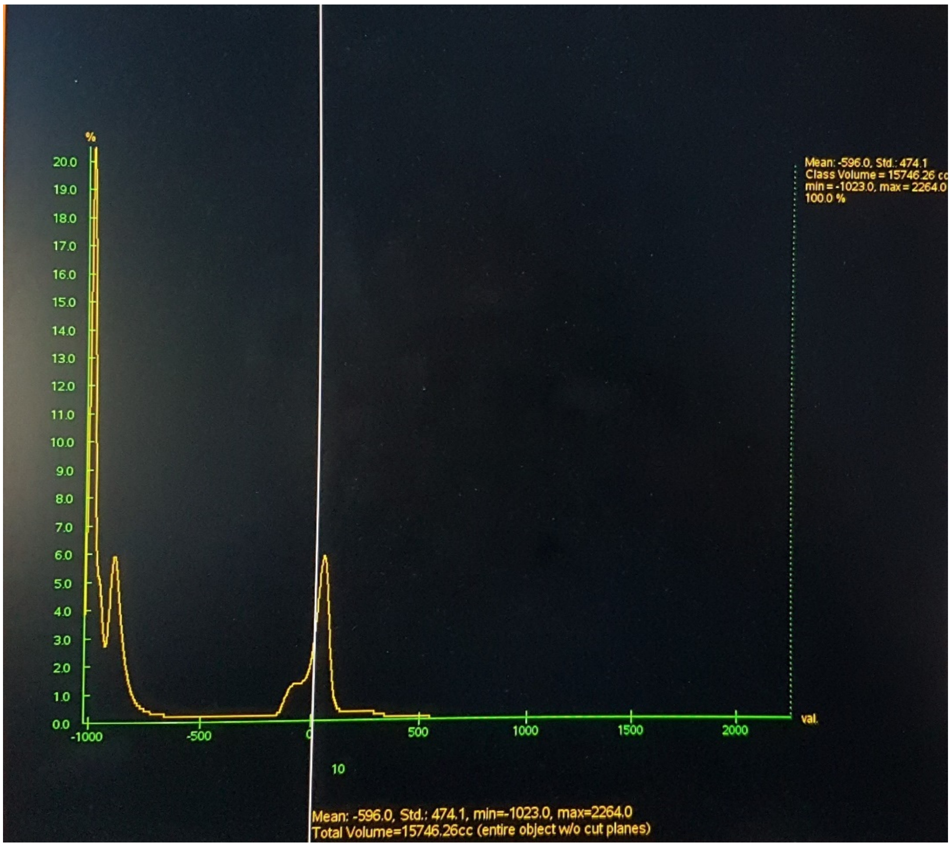

Figure S1. On the volume viewer mode, a histogram depicting frequencies of the attenuation coefficients of all voxels included in the acquired images of the chest was constructed.

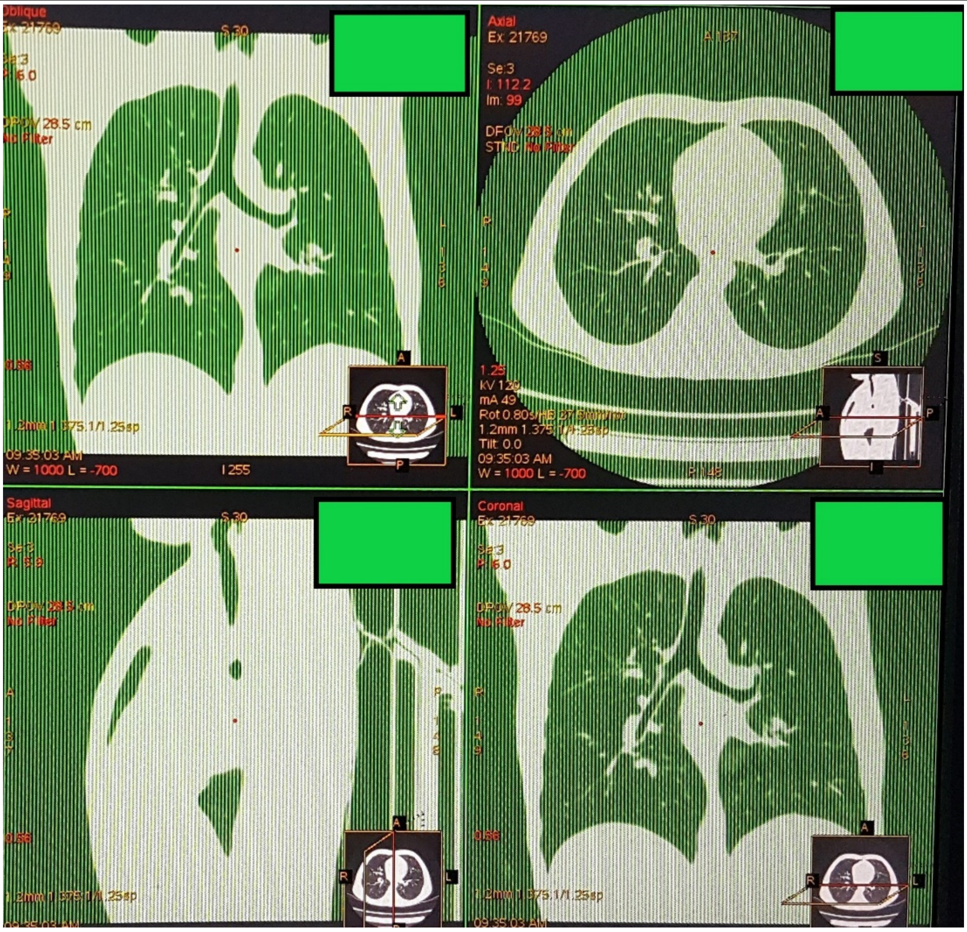

Figure S2. An axial, a sagittal and a coronal image of the chest were placed on the screen. Using two lines vertical to the x-axis of the histogram (attenuation coefficient values) an upper and a lower attenuation limit were set visually so that the lung parenchyma was visible in the three chest images (axial, sagittal and coronal).

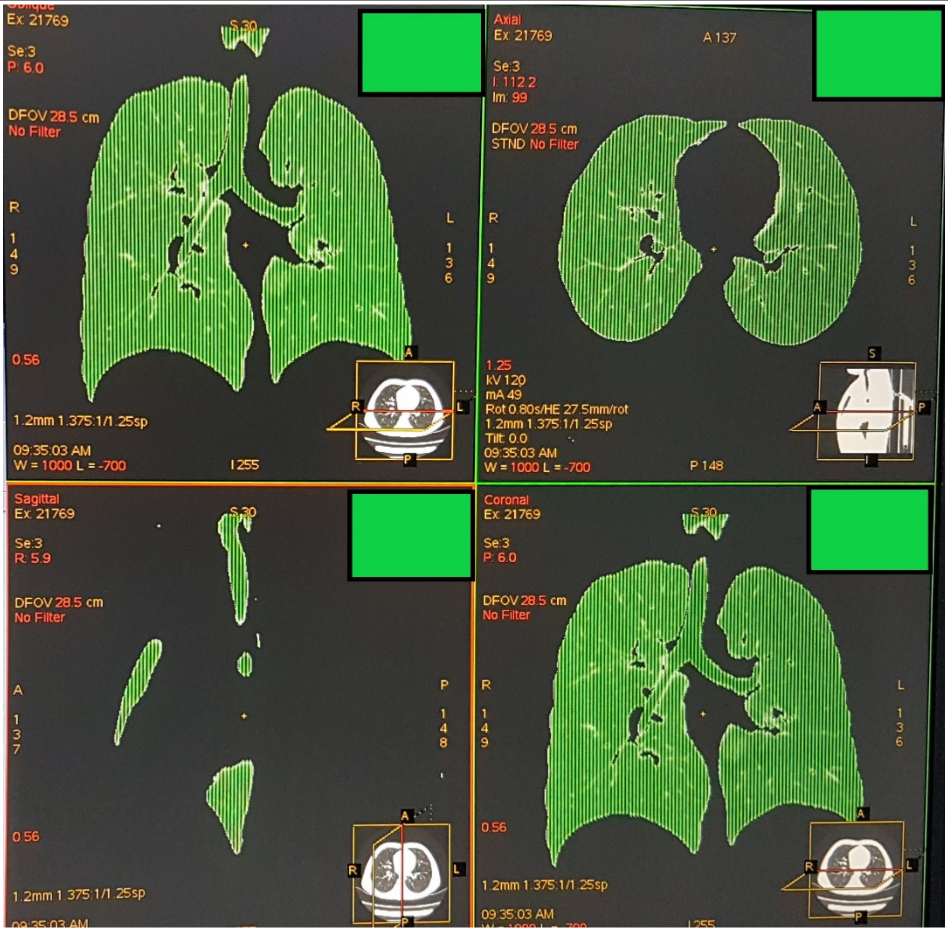

Figure S3. "Lung" was chosen as the imaging window so that other structures were not shown. Air located outside the chest (e.g. gastric air bubble) and outside the subject's body was still visible and included in the automatically derived attenuation measurements. Thus, it was removed manually and at the end of the process, only the lung parenchyma, the trachea and bronchi were depicted.

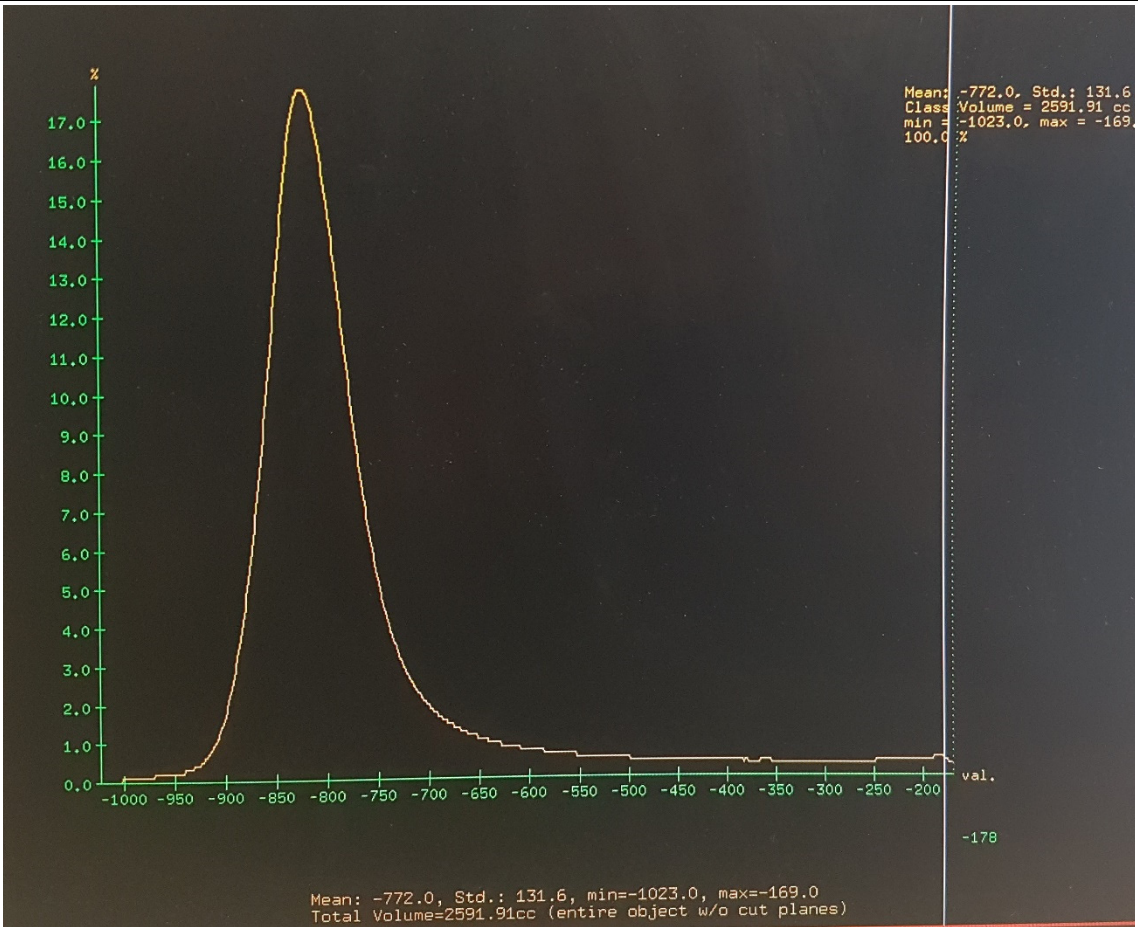

Figure S4. The total lung and airways volume, as well as the minimum attenuation value, maximum attenuation value, and the mean and standard deviation of attenuation values of all voxels included in the images of lung parenchyma and central airways were automatically provided by the software. Air contained in the central airways was trivial (less than 2%) compared to the total lung volume which was considered equivalent to total lung capacity (TLC_{CT}).

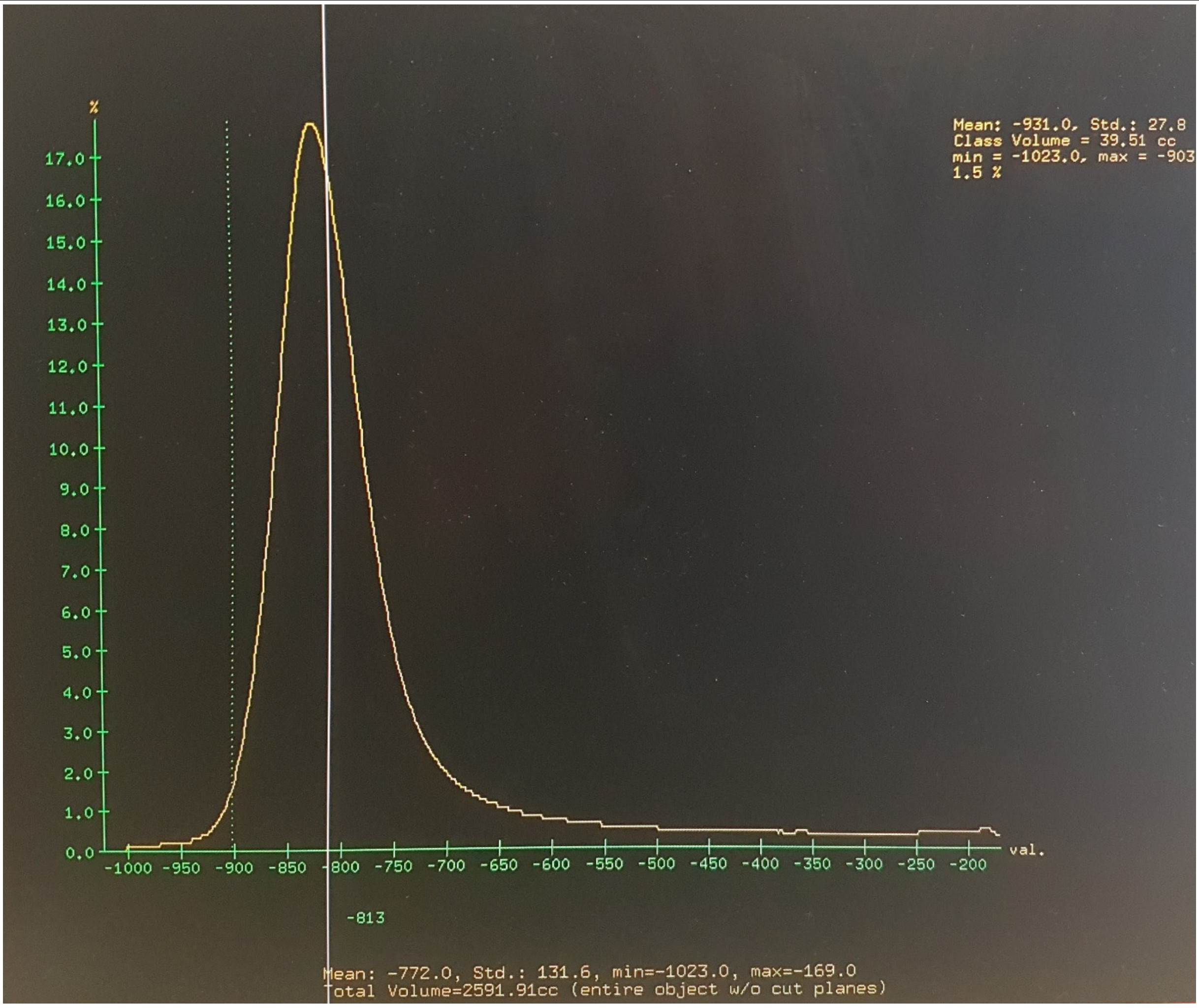

Figure S5. % total lung volume (%TLC_{CT}) with attenuation values < low-attenuation threshold (mean attenuation value - 1SD) was calculated.

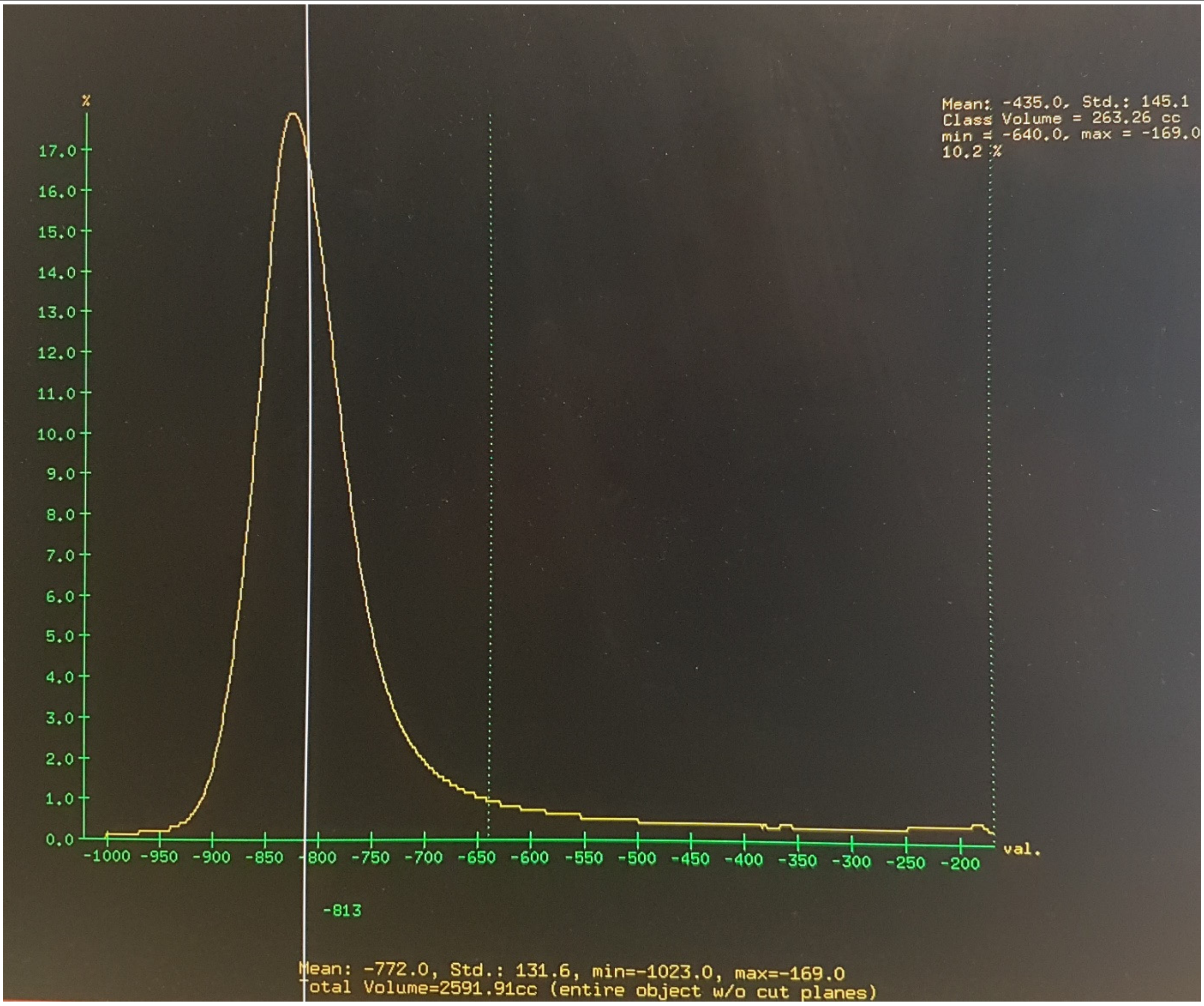

Figure S6. % total lung volume (%TLC$_{CT}$) with attenuation values > high-attenuation threshold (mean attenuation value +1SD) was also calculated.

Supplementary Results

Table S1. Study subjects' clinical diagnoses.

Diagnoses	N=80 (100%)
Acute lymphoblastic leukemia	7
Acute myelogenous leukemia	4
Adrenal tumor	2
Angiosarcoma	1
Aplastic anemia	1
Brain tumor	1
Cervical lymphadenopathy	3
Chest wall lesion	2
Chondrosarcoma	1
Diamond-Blackfan anemia	1
Ewing's sarcoma	11
Hepatosplenomegaly	1
Hodgkin's lymphoma	2
Langerhans cell histiocytosis	1
Mediastinal cystic hygroma	1
Mediastinal widening on chest X-ray	5
Nephroblastoma	3
Neuroblastoma	1
Non-Hodgkin's lymphoma	7
Osteosarcoma	11
Ovarian dysgerminoma	2
Possible foreign body aspiration	1
Possible hemoptysis	1
Retroperitoneal tumor	1
Rhabdomyosarcoma	2
Sarcoma	3
Testicular cancer	1
Thalassemia major	1
Trauma	2
